# The Influence of Removing the Ten-Minute Bout Requirement on National Physical Activity Estimates

**DOI:** 10.5888/pcd17.190321

**Published:** 2020-02-27

**Authors:** Emily N. Ussery, Kathleen B. Watson, Susan A. Carlson

**Affiliations:** 1 National Center for Chronic Disease Prevention and Health Promotion, Centers for Disease Control and Prevention, Atlanta, Georgia

## Abstract

The updated Physical Activity Guidelines for Americans (2nd edition) removes the requirement that physical activity should occur in bouts lasting 10 minutes or more to count toward the minimum aerobic guideline. Using self-reported data from the 2017 Behavioral Risk Factor Surveillance System (N = 386,960), we examined differences in national physical activity estimates with and without this requirement. Overall, 1.9% of adults reported activities in short bouts (<10 minutes). When excluding short bouts, 29.9% were inactive, 20.7% were insufficiently active, and 49.4% were active. When including short bouts, 29.1% were inactive, 21.4% were insufficiently active, and 49.5% were active. Eliminating the 10-minute-bout requirement had little effect on physical activity estimates.

**Summary**

**What is already known on this topic?**

Physical Activity Guidelines for Americans, 2nd edition*,* asserts that any duration of physical activity provides health benefits, removing the previous requirement that activity should be accumulated in bouts lasting 10 minutes or more.

**What is added by this report?**

Our report indicates that including short bouts (less than 10 min) of self-reported physical activity in total volume of self-reported physical activity has minimal impact on national physical activity estimates.

**What are the implications for public health practice?**

The updated guidelines might necessitate changes to definitions and measures used in public health surveillance. Our findings suggest the change in surveillance measures to accommodate the elimination of the 10-minute-bout requirement is unlikely to markedly influence national prevalence estimates obtained from the Behavioral Risk Factor Surveillance System or similar surveys.

## Objective

The Physical Activity Guidelines for Americans, 2nd edition, states that adults should perform at least 150 minutes per week of moderate-intensity aerobic physical activity or 75 minutes per week of vigorous-intensity physical activity, or an equivalent combination of both for substantial health benefits (1). Any duration of moderate to vigorous intensity physical activity (MVPA) contributes to this goal, removing the previous requirement that physical activity must be accumulated in bouts of at least 10 minutes (2). Our goal was to examine how incorporating this change into surveillance measures might influence national estimates of physical activity levels. We used data from the Behavioral Risk Factor Surveillance System (BRFSS) to compare self-reported physical activity levels when short bouts were included or excluded from measures of total volume.

## Methods

We used self-reported data from the 2017 BRFSS, a state-based telephone survey of noninstitutionalized US civilians aged 18 or older. BRFSS survey design and methodology are described elsewhere (3). Data were collected from 450,016 respondents in 50 states, Guam, Puerto Rico, and the District of Columbia. The median response rate for combined landline and cellular telephone respondents across states was 45.9% (range, 30.6%–64.1%).

Respondent characteristics were sex, age group (18–34, 35–49, 50–64, ≥65), race/ethnicity (non-Hispanic white, non-Hispanic black, Hispanic, non-Hispanic other, non-Hispanic multiracial), educational attainment (<high school graduate, high school graduate, some college, college graduate), and US Census region (Northeast, Midwest, South, West, US territories) (4). To assess physical activity, respondents reported the 2 nonoccupational, aerobic physical activities they spent the most time performing during the past month. For each activity, frequency (times per week or month) and bout duration (minutes or hours per bout) were reported. Each minute of vigorous-intensity physical activity was multiplied by 2, consistent with guidelines (1). Two measures of total physical activity volume were calculated according to the following guidelines: 1) minutes per week of MVPA, excluding bouts with reported duration less than 10 minutes or short bouts, according to the 2008 Physical Activity Guidelines for Americans (2), and 2) minutes per week of MVPA, including all reported bouts, as stated in the Physical Activity Guidelines for Americans, 2nd edition (1). For each measure of volume, respondents were categorized as inactive (no MVPA with a bout duration of 10 minutes or more or no MVPA), insufficiently active (some MVPA but not meeting the active definition), or active (150 minutes or more per week of MVPA).

We estimated the prevalence of reporting at least 1 physical activity occurring in short bouts overall and by respondent characteristics (sex, age group, race/ethnicity, educational attainment, census region). Differences in prevalence across characteristics were tested by using corrected Pearson χ^2^ tests, with the significance level at .05. We also estimated the prevalence of each physical activity level (inactive, insufficiently active, active) by using both measures of volume overall and by respondent characteristics. Respondents with missing demographics (n = 10,169) or physical activity (n = 52,887) were excluded (analytic sample = 386,960). Analyses were conducted in Stata 13.1 (StataCorp LP) by using weights and SVY procedures to account for the complex sampling design of BRFSS.

## Results

Overall, 1.9% of US adults reported at least 1 of their most common physical activities occurred in short bouts (Table 1). Significant differences in prevalence were observed by sex, race/ethnicity, educational attainment, and census region (*P* < .05). Overall, 29.9% of adults were classified as physically inactive and 20.7% as insufficiently active when excluding short bouts, compared with 29.1% and 21.4% when including all bouts (Table 2). The prevalence of being active was similar when excluding or including short bouts (49.4% vs 49.5%). Comparable findings were observed across categories of sex, age group, race/ethnicity, educational attainment, and census region.

**Table 1:** Prevalence of Reporting at Least One Short Bout of Physical Activity Among US Adults, 2017 Behavioral Risk Factor Surveillance System

Characteristic	Sample Size N^a^	Reported Short Bouts		*P* Value^c^
		n^a^	%^b ^(95% CI)	
**Overall**	386,960	6,879	1.9 (1.8–2.0)	—
**Sex**				
Male	170,546	3,267	2.0 (1.9–2.2)	.02
Female	216,414	3,612	1.8 (1.6–1.9)	
**Age group, y**				
18–34	62,933	1,028	1.9 (1.7–2.2)	.26
35–49	71,483	1,022	1.8 (1.5–2.1)	
50–64	117,297	1,978	1.8 (1.6–1.9)	
≥65	135,247	2,851	2.0 (1.9–2.2)	
**Race/ethnicity**				
Non-Hispanic white	298,919	5,359	1.9 (1.7–2.0)	.02
Non-Hispanic black	29,739	630	2.5 (2.0–2.9)	
Hispanic	31,787	445	1.7 (1.4–2.0)	
Non-Hispanic other	18,466	286	1.6 (1.0–2.2)	
Non-Hispanic multiracial	8,049	159	1.9 (1.4–2.5)	
**Educational attainment**				
Less than high school graduate	26,582	549	2.1 (1.7–2.4)	.02
High school graduate	103,462	1,909	2.0 (1.8–2.2)	
Some college	108,003	1,930	2.0 (1.8–2.2)	
College graduate	148,913	2,491	1.6 (1.5–1.7)	
**Census region**				
Northeast	64,512	1,326	2.5 (2.3–2.7)	<.001
Midwest	114,945	2,079	1.5 (1.4–1.6)	
South	116,005	2,147	2.3 (2.0–2.5)	
West	85,872	1,264	1.2 (1.0–1.3)	
US territories	5,626	63	0.5 (0.3–0.8)	

Abbreviation: CI, confidence interval.

a Unweighted counts. Respondents with missing data on demographic characteristics (n = 10,169) or physical activity (n = 52,887) were excluded from the analysis.

b Weighted prevalence of reporting at least 1 activity occurring in short bouts (<10 minutes). Respondents reported 2 types of nonoccupational physical activities or exercises, such as running, calisthenics, golf, gardening, or walking that they spent the most time doing in the past month. For each reported activity, frequency and bout duration were assessed by using the following questions: “How many times per week or per month did you take part in this activity during the past month?” and “When you took part in this activity, for how many minutes or hours did you usually keep at it?” Respondents who reported a bout duration of 1 to 9 minutes for either activity were categorized as reporting a short bout of physical activity.

c Corrected Pearson χ^2^ tests were used to examine differences across categories of demographic characteristics.

**Table 2:** Prevalence of Physical Activity Levels^a^ Among US Adults When Excluding or Including Short Bouts, 2017 Behavioral Risk Factor Surveillance System

Characteristic	2008 Physical Activity Guidelines^b^			Physical Activity Guidelines, 2nd Edition^c^		
	Inactive	Insufficiently Active	Active	Inactive	Insufficiently Active	Active
**Overall**	29.9 (29.6–30.3)	20.7 (20.4–21.0)	49.4 (49.0–49.7)	29.1 (28.8–29.4)	21.4 (21.1–21.7)	49.5 (49.1–49.8)
**Sex**						
Male	28.5 (28.0–29.0)	20.8 (20.4–21.2)	50.7 (50.2–51.2)	27.6 (27.1–28.0)	21.6 (21.2–22.0)	50.8 (50.3–51.3)
Female	31.3 (30.9–31.7)	20.6 (20.2–21.0)	48.1 (47.6–48.6)	30.6 (30.2–31.0)	21.2 (20.8–21.6)	48.2 (47.7–48.7)
**Age group, y**						
18–34	24.4 (23.8–25.0)	26.1 (25.5–26.7)	49.5 (48.8–50.2)	23.5 (22.8–24.1)	26.9 (26.3–27.6)	49.6 (48.9–50.3)
35–49	29.5 (28.8–30.2)	23.0 (22.4–23.6)	47.5 (46.7–48.2)	28.7 (28.0–29.4)	23.7 (23.1–24.3)	47.6 (46.9–48.3)
50−64	32.2 (31.6–32.8)	19.1 (18.6–19.6)	48.7 (48.1–49.4)	31.4 (30.8–32.0)	19.7 (19.2–20.2)	48.9 (48.2–49.5)
≥65	35.7 (35.1–36.3)	12.1 (11.7–12.5)	52.2 (51.5–52.8)	35.0 (34.4–35.6)	12.7 (12.3–13.1)	52.3 (51.7–53.0)
**Race/ethnicity**						
Non-Hispanic white	27.7 (27.4–28.1)	19.8 (19.5–20.1)	52.5 (52.1–52.8)	27.0 (26.7–27.3)	20.4 (20.1–20.7)	52.6 (52.2–53.0)
Non-Hispanic black	36.4 (35.4–37.5)	20.7 (19.8–21.6)	42.8 (41.7–43.9)	35.2 (34.2–36.3)	21.8 (20.8–22.7)	43.0 (41.9–44.1)
Hispanic	36.3 (35.2–37.3)	22.3 (21.3–23.2)	41.5 (40.4–42.6)	35.3 (34.3–36.4)	23.1 (22.2–24.1)	41.5 (40.5–42.6)
Non-Hispanic other	25.3 (23.7–26.9)	25.6 (23.9–27.2)	49.1 (47.3–51.0)	24.5 (22.9–26.1)	26.2 (24.5–27.8)	49.3 (47.5–51.2)
Non-Hispanic multiracial	26.6 (24.7–28.4)	19.4 (17.7–21.2)	54.0 (51.8–56.3)	26.0 (24.2–27.8)	19.9 (18.1–21.7)	54.1 (51.9–56.3)
**Educational attainment**						
Less than high school graduate	47.0 (45.8–48.1)	17.3 (16.4–18.3)	35.7 (34.6–36.9)	45.9 (44.7–47.1)	18.3 (17.4–19.3)	35.8 (34.6–36.9)
High school graduate	36.6 (35.9–37.2)	18.6 (18.1–19.2)	44.8 (44.1–45.4)	35.7 (35.0–36.3)	19.5 (18.9–20.0)	44.8 (44.2–45.5)
Some college	27.9 (27.3–28.5)	20.9 (20.4–21.5)	51.2 (50.5–51.8)	27.1 (26.5–27.7)	21.5 (21.0–22.1)	51.4 (50.7–52.0)
College graduate	17.9 (17.5–18.3)	24.0 (23.5–24.4)	58.1 (57.6–58.7)	17.3 (16.9–17.7)	24.5 (24.0–24.9)	58.2 (57.7–58.8)
**Census region**						
Northeast	29.6 (28.9–30.2)	20.1 (19.6–20.7)	50.3 (49.6–51.0)	28.5 (27.8–29.2)	21.1 (20.5–21.7)	50.4 (49.7–51.1)
Midwest	29.3 (28.8–29.8)	21.2 (20.8–21.7)	49.5 (49.0–50.0)	28.7 (28.2–29.2)	21.7 (21.2–22.1)	49.6 (49.1–50.1)
South	33.7 (33.1–34.3)	20.5 (20.0–21.0)	45.8 (45.2–46.5)	32.6 (32.0–33.2)	21.4 (20.9–22.0)	46.0 (45.4–46.6)
West	23.4 (22.7–24.0)	20.8 (20.1–21.5)	55.9 (55.1–56.7)	23.0 (22.3–23.7)	21.0 (20.3–21.7)	56.0 (55.1–56.8)
US territories	55.7 (53.8–57.5)	24.2 (22.6–25.8)	20.1 (18.6–21.6)	55.3 (53.4–57.2)	24.6 (23.0–26.2)	20.1 (18.6–21.6)

a All values expressed as percentage (95% confidence interval). Respondents reported 2 types of nonoccupational physical activities or exercises, such as running, calisthenics, golf, gardening, or walking, that they spent most time doing in the past month. For each reported activity, the frequency and bout duration were assessed using the following questions: “How many times per week or per month did you take part in this activity during the past month?” and “When you took part in this activity, for how many minutes or hours did you usually keep at it?” Total volume of moderate intensity equivalent physical activity (min/wk) was calculated as weekly frequency multiplied by bout duration, excluding and including short bouts. Each minute of vigorous-intensity physical activity was multiplied by 2. For each measure of volume, respondents were categorized as inactive (no MVPA with a bout duration ≥10 min or no MVPA), insufficiently active (some MVPA but not meeting active definition), or active (≥150 min/wk MVPA). Respondents with missing demographic characteristics (n = 10,169) or physical activity (n = 52,887) were excluded (analytic sample: n = 386,960).

b Excludes short bouts of physical activity.

c Includes short bouts of physical activity.

## Discussion

A small percentage of US adults reported physical activities occurring in short bouts. Moreover, including short bouts in measures of total physical activity volume did not result in meaningful differences in population-level physical activity levels, overall or across demographic groups. Our findings suggest a need to modify physical activity surveillance measures to remove the 10-minute bout requirement to align with recent guidelines, because the current measures used might have a minimal effect on national estimates of physical activity levels, particularly those obtained from BRFSS or similar surveys (1).

Our findings are inconsistent with those using device-based measures of physical activity. Studies using accelerometers found considerable variations in total volume of MVPA and adherence to guidelines when comparing activity accumulated in bouts of 10 minutes or more versus all accumulated activity (5,6). One possible explanation for the discrepancy is self-reported measures do not capture short bouts of physical activity as well as device-based measures because of the difficulty recalling short or unplanned episodes of physical activity (7). Moreover, the BRFSS questionnaire assesses average bout duration of respondents’ top 2 physical activities, whereas accelerometers capture individual bouts of activities.

Our study’s findings might not generalize to other surveillance systems that collect physical activity data. Although BRFSS captures physical activity that occurs in short bouts, 2 other major US surveillance systems exclude short bouts as part of their survey questions. In the National Health and Nutrition Examination Survey (NHANES), respondents report physical activity performed for at least 10 minutes continuously (8). Similarly, the National Health Interview Survey (NHIS) asks about activities respondents perform for at least 10 minutes (9). The effects of removing the clause on physical activity estimates derived from NHANES or NHIS is unknown. Future studies can be undertaken to examine any potential effects more closely.

This study has limitations. First, BRFSS data are self-reported and could be subject to social desirability or recall biases. Second, respondents report only their top 2 nonoccupational physical activities. Thus, total physical activity, particularly activities occurring in short bouts, might be underestimated. Third, the median response rate was 45.9%; lower response rates could result in response bias, although BRFSS weighting and survey methodology are designed to adjust for nonresponse, noncoverage, and undercoverage issues (10). The study also had strengths, including the large national sample and data from the only US surveillance system that assesses short bouts of physical activity.

Accurate public health monitoring of behaviors relies on consistent definitions and measures over time; however, new guidelines can necessitate changes to how outcomes are defined and measured. Our findings suggest that removal of the 10-minute bout requirement to align with updated physical activity guidelines is unlikely to markedly influence national estimates of physical activity obtained from BRFSS or similar surveys.
